# Selective Listening to Unpredictable Sound Sequences Increases Tonic Muscle Activity in the Human Vestigial Auriculomotor System

**DOI:** 10.1523/ENEURO.0301-25.2025

**Published:** 2025-10-17

**Authors:** Adrian Mai, Steven A. Hillyard, Daniel J. Strauss, Farah I. Corona-Strauss

**Affiliations:** ^1^Systems Neuroscience and Neurotechnology Unit, Faculty of Medicine, Saarland University & htw saar, Homburg/Saar 66421, Germany; ^2^Center for Digital Neurotechnologies Saar, Faculty of Medicine, Saarland University, Homburg/Saar 66421, Germany; ^3^Department of Neurosciences, School of Medicine, University of California San Diego, La Jolla, California 92093; ^4^Leibniz-Institute for Neurobiology, Magdeburg 39118, Germany

**Keywords:** auditory attention, electromyography, posterior auricular muscle (PAM), selective attention, vestigial auriculomotor system

## Abstract

Recent investigations have revealed that selective attention to lateralized speech increases ipsilateral tonic electromyographic activity in the vestigial human auriculomotor system. However, it has yet to be determined whether this modulation depends upon predictive cues that are inherent in continuous speech or whether it is a general concomitant of selective attention to sounds in the auditory periphery. The present study addressed this question by replacing speech with randomized, unpredictable sequences of brief tonal stimuli in a dichotic listening task that necessitated a sustained anticipatory focus of attention. Participants (8 female, 23 male) were presented with sequences of brief tone bursts in one ear and frequency-modulated “chirps” in the other ear and were instructed to focus on sounds in one ear and report attenuated deviant stimuli in that ear. Posterior auricular muscle (PAM) activity was recorded behind both ears, and non-rectified stimulus-locked responses were assessed to ensure the reliability of PAM activity. Recordings of non-stimulus-locked rectified activity indicated that ipsilateral tonic PAM amplitudes were elevated when same-side sounds were attended, and follow-up analyses demonstrated that these modulations were independent of sound-evoked PAM reflexes. These findings provide evidence that this ipsilateral tonic increase in PAM activity is generally present in scenarios of lateralized selective listening and not reliant on predictive linguistic cues that may facilitate tracking of the attended stream. Due to its accessibility and capability of decoding the spatial focus of attention, this PAM modulation could support the development of intelligent hearing devices that maximize sensitivity toward a user’s listening goals.

## Significance Statement

This study demonstrates that ipsilateral tonic activity of the posterior auricular muscle (PAM) is elevated when lateralized sounds are selectively attended, even when stimulation sequences have an intermittent and unpredictable structure that requires a sustained, non-predictive mode of attending. This represents an important extension of previous studies that revealed similar modulations during listening to continuous speech and suggests that the tonic modulation of PAM activity is generalizable across different scenarios of selective listening. Additionally, the present findings support the emerging view that the human auriculomotor system, despite its vestigial expression, operates in a remarkably nuanced manner. Integrating the observed modulations into intelligent hearing devices could provide an easily accessible means for decoding the lateralized focus of endogenously directed auditory attention.

## Introduction

The human auriculomotor system has been hypothesized to represent the “neural fossil” of a vestigial pinna-orienting system (Hackley, 2015; Strauss et al., 2020), and even though evolutionary processes may have diminished the overt expression of this mechanism (Hackley, 2015), it has been shown to be still visible to the naked eye in some individuals (Strauss et al., 2020). Recent studies have shown that electromyographic (EMG) activity of the auricular muscles can be reliably recorded from around and inside the ears (Schroeer et al., 2023) and might be utilized for the development of smart technologies (Liugan et al., 2018) such as intelligent hearing devices that optimize the users’ listening experience according to their endogenous intentions as decoded from electrophysiological signals (Strauss et al., 2020; Schroeer et al., 2023). While related research has predominantly relied on electroencephalographic (EEG) recordings (Fiedler et al., 2017; Geirnaert et al., 2021; Vandecappelle et al., 2021), there is emerging evidence that auriculomotor information could contribute valuable information about the direction of a person’s endogenous auditory attention.

Specifically, Strauss et al. (2020) presented participants with two different spoken stories that were spatially separated in the azimuth plane and instructed them to selectively attend to one of the stories. Their results showed that the tonic EMG activity levels of several peri-auricular muscles including the posterior, superior, and anterior auricular muscles (PAM/SAM/AAM) were consistently higher on the side of the head toward which attention was directed. Conceptually, this dual-speaker paradigm was similar to the classic dichotic listening task used by Hillyard et al. (1973) to investigate the effects of selective auditory attention on event-related potentials, in which participants were presented with unpredictable sequences of brief stimuli (tone bursts) to each ear at random with the instruction to attend to one ear at a time and to detect occasional deviant target stimuli in the attended stream. Using a similar design, Hackley et al. (1987) demonstrated that selective attention to tones in one ear enhanced the short-latency (5–30 ms) PAM reflexes (see O’Beirne and Patuzzi, 1999 for a review on the PAM reflex) on the side of the head where attention was directed. However, since Hackley et al.’s study only investigated stimulus-evoked PAM activity, it remains unclear whether tonic modulation of PAM activity like that observed by Strauss et al. (2020) is exclusive to scenarios of selective listening to lateralized speech, where attention may be facilitated through predictive cues (Cutler et al., 1997; Friederici, 2002), or whether such tonic modulation also occurs in more general situations such as when attention is directed in a sustained, non-predictive manner to intermittent and randomized sound sequences.

**Figure 1.**
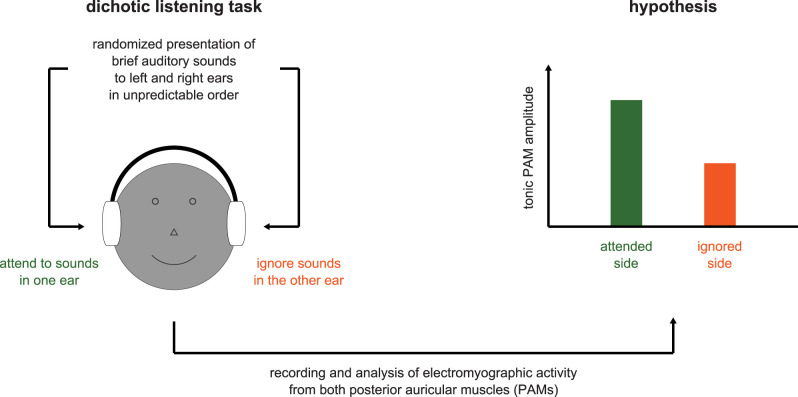
Schematic overview of the study and the research hypothesis. Participants carried out a dichotic listening task in which they were presented with unpredictable sequences of brief auditory sounds to each ear (tone bursts in one ear and chirps in the other ear) and were instructed to attend to one ear at a time. Their behavioral task was to detect occasional attenuated deviant stimuli in the attended stream. Throughout the experiment, electromyographic activity was recorded from the surface of the left and right posterior auricular muscles (PAMs). These data were analyzed to investigate the hypothesis that sustained anticipatory selective attention would increase the tonic PAM amplitude on the attended side.

To address this question, the present study revisited the classic dichotic listening scenario with a focus on investigating tonic PAM activity. Figure 1 provides a schematic overview of the experiment and the research hypothesis. Participants were repeatedly presented with sequences of brief, intermittent stimuli that were delivered in unpredictable order to one ear or the other at random with the task of attending to the sounds in only one ear at a time and reporting occasional attenuated deviant stimuli (targets) in the attended stream. Instead of delivering pure tones that differed in pitch between the ears, as was done by Hackley et al. (1987), however, tone bursts were only presented to one ear, and the competing stream in the other ear consisted of frequency-modulated “chirps,” which are known to evoke well-defined PAM reflexes (Agung et al., 2005). Throughout the experiment, surface-EMG recordings were obtained from each PAM, and rectified waveforms were analyzed to investigate the hypothesis that sustained selective attention would increase the tonic PAM amplitude on the attended side. Importantly, the present design with discrete randomized stimuli facilitated investigation of whether directed attention produces a tonic PAM increase that persists during inter-stimulus intervals rather than being driven by continuous, predictive stimulation such as speech. Such lateralized tonic activity would increase the utility of the PAM for indicating the direction of sustained, anticipatory attention prior to stimulus delivery. As a preview, the experimental results fully supported our hypothesis and suggest that tonic PAM amplitudes reliably indicate the side of attended auditory stimuli in the absence of any cues that may facilitate the prediction of the stimulation sequence structure.

## Materials and Methods

Some information on the present study (see sections Participants, Stimuli, Experimental design, and Data acquisition) was reported in a previous publication of electrophysiological data from the same experiment that was focused on effects of selective attention on auditory event-related potentials elicited by chirp and tone burst stimuli that were recorded in parallel with the PAM activity (Strauss et al., 2025). Starting with section EMG pre-processing, all information is original to the present analyses.

### Participants

Thirty-one volunteers (8 female, 23 male) with a mean age of 26.8 ± 3.7 years (mean ± standard deviation) from the university environment participated in the study. Inclusion criteria were no history of neurological diseases as assessed via self-report and normal hearing as confirmed by a mean hearing level of <15 dB sound pressure level (SPL) across all frequencies tested via pure-tone audiograms. Prior to audiogram registration, participants were informed about the experimental paradigm, which was approved by the local ethics committee (Ärztekammer des Saarlandes, Saarland Medical Council; identification number: 305/20) and accorded with the Declaration of Helsinki.

### Stimuli

The stimulus sequences comprised two types of brief auditory sounds that were digitized at 44,100 Hz and generated using MATLAB (The MathWorks). Stimuli in one ear were 800 Hz tone bursts with a total duration of 50 ms that included rise and fall times of 12 ms each, which were achieved by weighting the beginning and the end of the waveforms with the respective halves of a Hanning window of 24 ms duration. The competing stream in the other ear consisted of frequency-modulated chirp stimuli that were specified according to the A-Chirp (Fobel and Dau, 2004; Corona-Strauss et al., 2009) with a reference intensity level of 40 dB SPL, a duration of 14.9 ms, and a logarithmic increase in instantaneous frequency from 100 Hz to 9,800 Hz. In contrast to the tone burst stimuli, the chirp waveforms remained unwindowed to preserve their precisely shaped morphologies. Using these two types of stimuli was aimed at enabling a comparison of the effects of selective attention on the auditory event-related potentials elicited by tone bursts and chirps, as reported by Strauss et al. (2025). Specifically, the chirps were expected to provide major benefits for investigating such effects in the auditory brainstem response, as the design of the A-Chirp is optimized for eliciting these very early responses due to the synchronized neural firing that it produces along a large portion of the basilar membrane (Fobel and Dau, 2004). Additionally, chirp stimuli have been shown to produce larger PAM reflexes compared to other brief stimuli at equivalent hearing levels (Agung et al., 2005). However, it is unclear whether tone bursts and chirps may elicit different time-locked responses apart from the PAM reflex and whether they may be differently affected by selective auditory attention, and the present experimental design facilitated future investigations of such effects.

Tone bursts and chirps were used to generate ten dichotic stimulation sequences in which the two types of stimuli were assigned to different ears. Within each sequence, successive stimulus events were assigned to one ear or the other at random with randomized inter-stimulus intervals of 250–400 ms between any two events. Furthermore, in each ear, infrequent deviant stimuli occurred every 3–20 events with a probability of 9.4%, with waveforms that were identical to the standard waveforms but attenuated by 15 dB SPL. These fainter stimuli served as targets for response in the attended ear. The number of sequences in which each type of stimulus was presented to each ear was evenly balanced. On average, each sequence delivered 300 stimuli per ear while lasting 3.26 min.

All stimuli were presented using circumaural headphones (HDA300, Sennheiser) and routed via a digital audio workstation (Studio One, PreSonus) and an external audio interface (Scarlett 18i20, Focusrite). For each participant, the hearing thresholds were determined for both types of stimuli, and the playback intensity level of the respective channel in each stimulation sequence was set to an intensity of 40 dB sensation level (SL) for standard stimuli. This procedure yielded mean stimulus intensities across participants and ears of 59.5 dB SPL for chirps and 52.1 dB SPL for tone bursts. Due to their different sound characteristics, the playback intensities were afterwards fine-tuned to achieve an overall uniform perception of loudness. As the stimulus intensities were already pre-matched in terms of SL and substantial differences in loudness perception were not expected across the homogeneous participant group, the maximal fine-tuning adjustment was limited to ±3 dB SPL.

### Experimental design

Participants were presented with the dichotic sequences of tones in one ear and chirps in the other and were cued to direct attention toward one ear at a time and to signal their detections of deviant targets via button presses. A press within 50–1,500 ms after a target was considered a correct detection. This procedure was repeated for 20 listening trials that were presented in pseudo-randomized order with ten sequences each under attend-left and attend-right conditions. Consequently, the paradigm yielded five trials for each combination of the factors of stimulus type (chirps or tones), stimulus location (left or right side), and attended location (left or right side).

Prior to the main experiment, participants underwent a short training session to familiarize themselves with the procedure. Throughout the study, they were sitting in a comfortable armchair and fixating their gaze on a small sphere that was positioned at 2 m distance at sight level.

### Data acquisition

Data acquisition and processing were coordinated using MATLAB and Simulink (The MathWorks). Two non-recessed Ag/AgCl electrodes (BME-6, BioMed Products) were placed on each PAM (Fig. 1*B* in Strauss et al., 2020) and referenced against an additional ground electrode at the center of the upper forehead to record unipolar surface-EMG signals. All data were digitized with a biosignal amplifier (g.HIamp, g.tec medical engineering) at a sampling rate of 9,600 Hz along with trigger signals that indicated stimulation events. Sensors were prepared with electrode cream, and during trial breaks impedances were controlled to be lower than 10 kΩ. Additional EEG recordings were simultaneously obtained from passive electrodes on the mastoid and vertex positions as well as from a 128-channel EEG cap equipped with active sensors (g.SCARABEO, g.tec medical engineering), but these data (reported in Strauss et al., 2025) were not considered in the present analyses of PAM activity.

### EMG pre-processing

Continuous bipolar EMG recordings from each PAM were obtained by calculating the difference potential between the two associated unipolar channels and resampled to 4,800 Hz including an anti-aliasing filter (*resample.m* with default parameters). These signals were filtered with a comb filter to remove power line noise at 50 Hz and all of its harmonics (*iircomb.m* with Q factor of 35) as well as with a 20–1,000 Hz bandpass filter of 4800th order (*fir1.m*), both with forward and backward passes to avoid phase distortions (*filtfilt.m*).

### Participant selection

Prior to the main analyses of tonic amplitudes obtained from rectified PAM recordings, a participant selection procedure was carried out to ensure that only those that showed signatures of reliable PAM activity were included in the final investigations. Due to a lack of clear, characteristic features in the continuous EMG activity that may be suited for evaluating the signal reliability, these analyses were based on screenings of time-locked PAM responses to the chirp and tone burst stimuli that were extracted from the non-rectified EMG recordings. The time-locked responses were expected to encompass the PAM reflex with its stereotypical waveform, which would provide an easily accessible feature to evaluate the overall reliability of each participant’s data set. The detailed processing steps for the participant selection procedure are described in sections Processing of time-locked PAM responses and Reliability analysis of time-locked PAM responses; to skip these details the reader may proceed to the major analyses of the tonic PAM activity in section Analyses of tonic PAM activity.

#### Processing of time-locked PAM responses

Time-locked PAM responses were first obtained in response to the chirp stimuli. To this end, single-trial responses to all chirps were extracted from the pre-preprocessed recordings with 1,000 ms pre-stimulus and post-stimulus periods relative to stimulus onsets, and, separately for standard and deviant stimuli, grouped in a 2 × 2 × 2 design according to the factors of PAM location (left or right side), chirp location (left or right side), and attended location (left or right side). Due to the spectrotemporal characteristics of chirps, the peak latencies of chirp-evoked PAM reflexes are delayed by the stimulus duration (Agung et al., 2005), i.e., the timing of the stimulus that triggers the PAM response (*t* = 0 ms) corresponds to the highest frequency component of the chirp at its offset. The time bases of the extracted epochs were consequently shifted to a “time-zero” at chirp offset, and the waveforms were linearly detrended and baseline-corrected by subtracting the mean amplitude between −250 and 0 ms from each point in the PAM response waveform.

Segmentation of the time-locked epochs was followed by an artifact rejection procedure that was carried out for separately for standard and deviant stimuli. For each participant and single-trial of the 2 × 2 × 2 design, the signal-to-noise ratio (SNR) was calculated as the ratio of the variances in the 250 ms windows immediately after—which was expected to encompass the PAM reflex—and before chirp offset (“time-zero”) and transformed to decibel ( $10\log_{10}$). While low SNRs would likely be indicative of noisy data segments, extremely high SNRs could possibly result from excessive noise in post-stimulus periods that is present in addition to the PAM reflex. Thus, single-trial epochs were only retained if their SNR deviated not more than three standard deviations from the overall mean across all single-trials of the 2 × 2 × 2 design. This step was repeated for each participant, and the smallest count of artifact-free epochs across cells and participants was identified as the number of epochs to include for each cell. Finally, and for each participant, the specified number of single-trials (1,187 for standard and 111 for deviant chirps) with SNRs closest to the previously calculated overall mean SNR were identified for each cell as the most representative responses and selected for further analysis. The 2 × 2 × 2 designs for standards and deviants were afterwards divided with respect to the factor of PAM location, resulting in separate 2 × 2 designs for the left and right PAM with the factors of chirp location (left or right side) and attended location (left or right side) that were used to investigate effects for both muscles separately.

Amplitude comparisons of time-locked responses between left and right PAMs can be complicated by a variety of factors. These pitfalls include, for example, the well-known variability in PAM activity within and between individuals (Picton et al., 1974) and the fact that visual identification of the optimal electrode locations for recording can be challenging. To facilitate the comparability of time-locked PAM responses between left and right muscles, response amplitudes were normalized based on the PAM reflex. Specifically, this method aimed for normalizing the amplitudes of averaged PAM reflex waveforms, which served as robust normalization tools due to their high SNR with respect to the averaged EMG background activity. The normalization procedure was carried out separately for each PAM and participant and included three steps that were designed to preserve the relative differences between all responses recorded from each PAM. First, the single-trial epochs were averaged for each cell of the 2 × 2 designs for standard and deviant stimuli. Second, the averaged waveforms were pooled across standard and deviant stimuli, and the global normalization factor was identified as the maximum absolute amplitude across all eight waveforms in a typical PAM reflex peak latency range of 5–30 ms (Hackley et al., 1987; O’Beirne and Patuzzi, 1999). Finally, all single-trials for standard and deviant stimuli that contributed to the calculations in the first step were divided by the normalization factor.

The processing of time-locked PAM responses was equivalently repeated for the tone bursts. In this case, identified counts of artifact-free single-trials were 1,365 for standard tones and 149 for deviant tones. For these responses, onsets (*t* = 0 ms) were adjusted to the terminations of tone burst rise times, i.e., the time points at which the tone bursts reached maximum amplitude.

#### Reliability analysis of time-locked PAM responses

The time-locked PAM responses elicited by chirp and tone burst stimuli were separately screened to evaluate the overall trustworthiness of each participant’s data set. Specifically, a reliability testing procedure was implemented via correlation-based half-split (odd vs even) checks of PAM reflex waveforms, which, in addition to their previous use for the normalization strategy, provided robust correlation tools due to their stereotypical morphology. To improve the stability of these tests, they were solely based on responses to standard stimuli, for which the number of available single-trials was larger by an order of magnitude compared to the deviant stimuli. For each cell of the 2 × 2 designs for the left and right PAM, the single-trial epochs were split into two sets of odd- and even-indexed responses, waveforms within each set were averaged, and the Pearson correlation coefficient between the two averaged responses was calculated during the PAM reflex latency range of 5–30 ms for each of the eight cells. The resulting correlations were pooled across cells, and the reliability criterion was set to a correlation of 0.4 or higher for at least six out of eight (75%) cells. Using this threshold, data were only considered to be reliable if both PAMs passed the half-split checks in at least 50% of their conditions, ensuring that both muscles provided trustworthy EMG recordings.

For chirp stimuli, the reliability criterion was met by 17 out of 31 participants (54.8%). Conversely, tone bursts failed to reliably elicit PAM reflexes for all participants, suggesting that the combination of the slow rise to full intensity and their rather low intensity level failed to evoke PAM reflexes on a regular basis. Ultimately, only the set of 17 participants that passed the reliability tests for chirps were selected for subsequent analyses of tonic PAM activity.

### Analyses of tonic PAM activity

Analyses of tonic PAM activity are commonly based on normalized amplitudes of ongoing rectified PAM activity (Strauss et al., 2020; Schroeer et al., 2025), i.e., estimates of instantaneous amplitude obtained via demodulation of PAM spike trains. To facilitate direct comparisons between previously reported modulations and any effects that may be observed in the present study, the primary tonic analysis was implemented using a similar approach that considered continuous PAM activity over extended periods of time (see section Analysis of tonic effects in ongoing PAM activity). A limitation of this strategy is that it does not provide a clear delineation between observations of enhanced PAM amplitudes that result from higher-amplitude time-locked activity on one hand, and an increased sustained muscle tone on the other. While this may be counteracted to at least some extent by blanking the PAM reflex intervals in the ongoing activity, there would be still some ambiguity regarding the precise timing of the reflexive responses. Apart from that, additional components in the time-locked PAM responses, such as those that have been recorded in response to naturalistic auditory stimuli presented at unpredictable locations (Strauss et al., 2020; Schroeer et al., 2023, 2024), could further confound the results.

A more elegant approach that bypasses the addressed caveats can be implemented by picking up on the time-locked analyses reported in section Participant selection. In principle, if any amplitude effects observed for the primary tonic analysis would result from true modulations of sustained PAM activity, these effects would be expected to be also present in the pre-stimulus periods of artifact-free rectified PAM responses that were elicited by the chirps. Such an analysis of pre-stimulus PAM activity, which allows for a clear separation between attention effects on rectified tonic PAM amplitudes and time-locked PAM responses, was carried out as the secondary tonic analysis (see section Analysis of tonic effects in time-locked PAM activity).

#### Analysis of tonic effects in ongoing PAM activity

For the primary tonic analysis based on ongoing PAM activity, the root-mean-square (RMS) envelope was calculated for each pre-processed EMG recording using a sliding window of 1 s duration (*envelope.m*). All envelopes were then trimmed to a region of interest that lasted for 180 s and started 5 s after the first stimulus event onset in each trial. These data were segmented into five consecutive, non-overlapping epochs of 36 s duration, and the mean RMS amplitude was calculated for each of the segments. For each PAM and participant, all amplitude values were afterwards pooled, and values that did not fall within the range between the 2.5th percentile and the 97.5th percentile were identified as outliers and removed. The remaining epoch amplitudes were normalized to *z*-scores within each PAM and participant, which importantly preserved all relative effects of attention. Normalized tonic PAM amplitudes were then categorized with respect to the side of the PAM from which they were recorded (left or right PAM), the type of stimulus that was presented to that side (tone bursts or chirps), and the side that attention was directed to (left or right side). For each cell of the categorization scheme, the values for each of the segments were finally averaged across the associated listening trials, which resulted in a data set with dimensions 2 × 2 × 2 × 5 (left and right PAM × tone bursts and chirps × attend-left and right × five segments over time) for each participant.

#### Analysis of tonic effects in time-locked PAM activity

For the secondary tonic analysis based on time-locked PAM activity elicited by chirps, RMS envelopes of pre-processed signals were additionally calculated using a short sliding window of 10 ms, which ensured that stimulus-locked response components such as the PAM reflex remained localized in time. Similar to the segmentation of non-rectified recordings (see section Processing of time-locked PAM responses), the resulting envelopes were epoched into single-trial responses to all chirps that were time-locked to chirp offsets (“time-zero”) with pre-and post-stimulus periods of 100 ms each, and all single-trials that corresponded to the previously identified artifact-free (non-rectified) epochs were selected for standard and deviant chirps. These epochs were grouped according to a 2 × 2 × 2 × 2 design with factors of PAM location (left or right side), chirp location (left or right side), attended location (left or right side), and the type of chirp (standard or deviant). Since this time-locked analysis solely aimed to examine tonic pre-stimulus activity (preceding the PAM responses to standard and deviant chirps) for each PAM location (left or right side) and according to the listening condition (direct attention toward or away from side of PAM), epochs were subsequently combined for left and right chirp locations and averaged across single-trials. The resulting 2 × 2 × 2 categorization was then separated into left and right PAM locations, yielding separate 2 × 2 designs for each PAM with the factors of attended location (left or right side) and the type of chirp (standard or deviant). Separately for each PAM and participant, the amplitudes of averaged waveforms were then normalized across all four cells of the 2 × 2 design as described in the following: in contrast to the processing of non-rectified PAM responses, the subtractive baseline normalization was now omitted, and the amplitude normalization was focused on detecting any differences in amplitude levels during the pre-stimulus baseline period between −100 and 0 ms. As the smallest inter-stimulus interval was 250 ms, this approach ensured that any attention-related differences in pre-stimulus amplitude levels would be independent of PAM reflexes. Specifically, amplitudes of averaged rectified PAM response waveforms for each 2 × 2 design were normalized via *z*-scoring based on the mean and standard deviation of the pre-stimulus amplitudes between −100 and 0 ms across all four averaged waveforms. This was achieved by, for each of the four averaged waveforms, subtracting the identified mean value and dividing the result by the identified standard deviation, which again preserved all relative differences due to attention effects within each PAM.

### Statistical analyses

Inferential statistics were mainly carried out to test for differences in normalized PAM amplitudes obtained from the primary tonic analysis of ongoing activity (see section Analysis of tonic effects in ongoing PAM activity). To this end, the data sets were reduced to dimensions 2 × 2 × 2 × 2 by retaining only the first and last time segments, thereby including amplitudes solely for the first and last trial segments. This differentiation was aimed at revealing any shifts in overall PAM activity that may have evolved over time (Strauss et al., 2020). The normalized PAM amplitudes were analyzed separately for the sides of tone burst and chirp delivery and subjected to separate factorial 2 × 2 × 2 repeated-measures ANOVAs (RMANOVAs) with within-subjects factors of PAM location (left PAM vs right PAM), listening condition (direct attention toward side of PAM vs direct attention away from side of PAM), and time (first trial segment vs last trial segment) as well as their interactions. RMANOVAs were implemented in R (v4.3.2; R Core Team, 2023) using the *afex* package (v1.3.0; Singmann et al., 2023). Additionally, pre-stimulus PAM amplitudes that resulted from the secondary tonic analysis of time-locked activity (see section Analysis of tonic effects in time-locked PAM activity) were analyzed using two-tailed within-subjects *t*-tests as reported in the Results. All tests were conducted with a significance threshold of *α* = 0.05.

## Results

**Figure 2.**
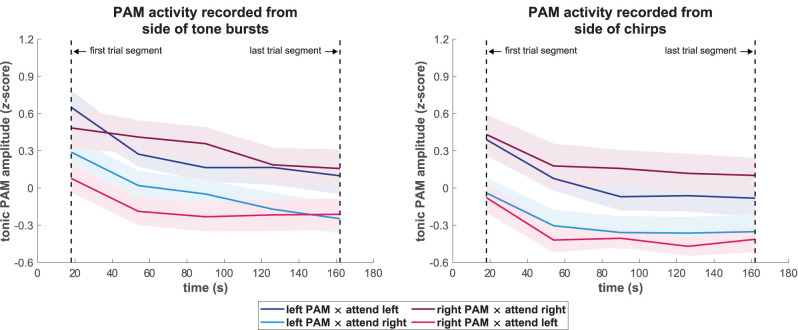
Time-resolved plot of tonic posterior auricular muscle (PAM) amplitudes during listening trials, categorized according to activity that was recorded from the side that was presented with tone bursts (left) and chirps (right). Mean amplitudes were calculated for five non-overlapping epochs of 36 s duration each over the trial duration of 180 s. Plotted values were assigned to segment centers. Solid lines represent the grand-average amplitudes across participants (N = 17) for the left and right PAM under attend-left and attend-right conditions, and shaded backgrounds show the associated standard errors. Most prominently, and consistently across the sides of presentation of tone bursts and chirp stimuli, tonic amplitudes for both PAMs were larger when attention was directed toward as opposed to away from their side of the head. Additionally, all waveforms presented a general decline in tonic amplitude over the course of the listening trials.

**Figure 3.**
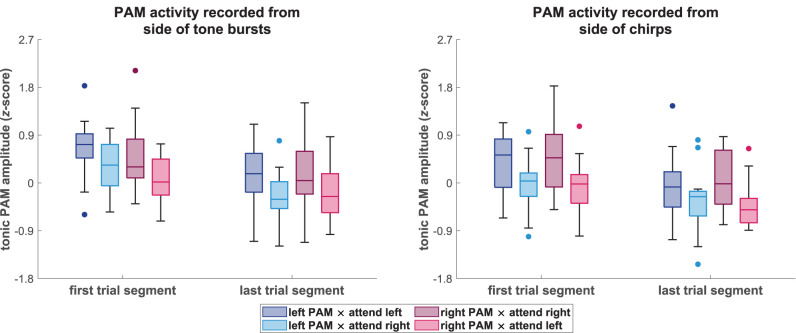
Distributions of tonic posterior auricular muscle (PAM) amplitudes for the first and last trial segments from the primary tonic analysis based on ongoing activity, categorized according to activity that was recorded from the side that was presented with tone bursts (left) and chirps (right). The plotted amplitudes correspond to the first and last segment of the time-resolved data shown in Figure 2 and were used to carry out statistical analyses via repeated-measures analyses of variance (RMANOVAs). Boxplots represent the amplitude distributions across participants (N = 17) for the left and right PAM under attend-left and attend-right conditions, and colored dots indicate values that deviated by more than 1.5 times the inter-quartile range from the lower or upper quartile. Consistent with the time-resolved plots shown in Figure 2, the tonic amplitudes for both PAMs were larger when attention was directed toward as opposed to away from their side of the head, independent of the type of stimulus that was presented to each side. Additionally, tonic amplitudes were consistently larger at the beginning as compared to the end of the listening trials.

Figure 2 presents the mean tonic PAM amplitudes from the analysis of ongoing activity (see Materials and Methods, section Analysis of tonic effects in ongoing PAM activity) across participants for each time segment over the course of the listening trials and for each combination of PAM location and attend-left/right condition, separately for the sides of presentation of tone burst and chirp stimuli. Overall, these time-resolved amplitude plots show patterns of effects that were consistent across both types of stimuli. Most notably, the tonic amplitudes for both PAMs were larger when stimulus sequences on their respective sides were attended compared to when they were ignored. Additionally, and regardless of the PAM location or the attended location, there was a general decline in tonic amplitude over the course of the listening trials. These observations were confirmed by the amplitude distributions across participants that are shown as boxplots in Figure 3 for the time intervals at the beginning and the end of the trials. Consistent with these observations, the 2 × 2 × 2 RMANOVAs that were carried out on the amplitudes shown in Figure 3 for both types of stimuli revealed a significant main effect for the factor of listening condition (*p* < 0.01 for both tone bursts and chirps). This result confirmed that directing attention to sounds in one ear produced enhanced tonic EMG activity in the ipsilateral PAM and emphasized the generalization of this effect across both ears and different types of brief stimuli. The factor of time was also significant (*p* < 0.01 for tone bursts and *p* < 0.05 for chirps), reflecting the reduced amplitude of PAM activity toward the end of the trial. Statistical details for all RMANOVA main effects are summarized in Table 1. Although the time-resolved plots in Figure 2 suggested that the overall amplitude separation between listening conditions was somewhat larger for the right PAM, the RMANOVA indicated that there was neither a significant main effect of PAM location for tone bursts (*p* = 0.61) nor for chirps (*p* = 0.83). Apart from these findings, none of the interaction effects were significant (all *p* ≥ 0.14 for tone bursts and *p* ≥ 0.29 for chirps).

**Table 1.** Summary of statistical details for repeated-measures analyses of variance (RMANOVAs) that were carried out for the tonic posterior auricular muscle (PAM) amplitudes shown in Figure 3

RMANOVA factors	Test statistic	Side of tone bursts	Side of chirps
	*F*(1, 16)	0.27	0.05
PAM location	*p*	0.61	0.83
	$\eta_{\;p}^{2}$	0.02	2.81 · 10^−3^
			
	*F*(1, 16)	11.86	11.60
Listening condition	*p*	3.34 · 10^−3^**	3.61 · 10^−3^**
	$\eta_{\;p}^{2}$	0.43	0.42
			
	*F*(1, 16)	12.34	7.83
Time	*p*	2.88 · 10^−3^**	0.01*
	$\eta_{\;p}^{2}$	0.44	0.33
			
	*F*(1, 16)	0.06	1.21
PAM location × Listening condition	*p*	0.81	0.29
	$\eta_{\;p}^{2}$	3.65 · 10^−3^	0.07
			
	*F*(1, 16)	2.41	0.40
PAM location × Time	*p*	0.14	0.53
	$\eta_{\;p}^{2}$	0.13	0.02
			
	*F*(1, 16)	0.04	0.91
Listening condition × Time	*p*	0.85	0.35
	$\eta_{\;p}^{2}$	2.45 · 10^−3^	0.05
			
	*F*(1, 16)	0.01	0.42
PAM location × Listening condition × Time	*p*	0.92	0.53
	$\eta_{\;p}^{2}$	6.54 · 10^−4^	0.03

Separate analyses were done for the PAMs on the sides of the head where tone bursts and chirps were presented. The table provides *F*-values, *p*-values, and standardized effect sizes as partial eta-squared ( $\eta_{\;p}^{2}$) for the within-subject factors of PAM location (left PAM vs right PAM), listening condition (direct attention toward side of PAM vs direct attention away from side of PAM), and time (first trial segment vs last trial segment) as well as their interactions. Asterisks denote statistical significance at different significance levels (*p < 0.05 and **p < 0.01).

**Figure 4.**
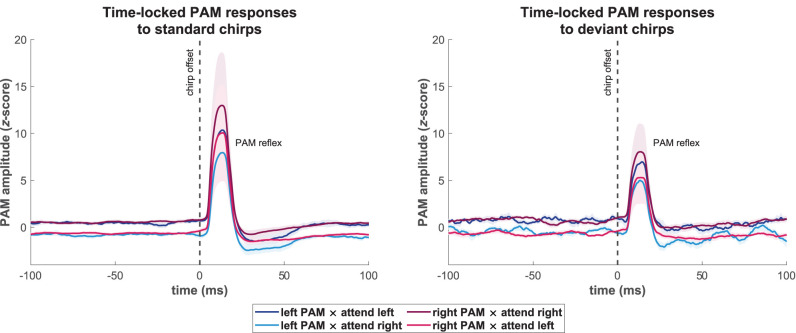
Rectified posterior auricular muscle (PAM) response (PAM) waveforms elicited by chirp stimuli from the secondary tonic analysis based on time-locked activity, categorized into responses to standard (left) and deviant (right) chirps. Amplitudes were normalized with respect to the pre-stimulus period between −100 and 0 ms, ensuring that baseline amplitude differences were not influenced by PAM reflex amplitudes. Solid lines represent the grand-average waveforms across participants (N = 17) for the left and right PAM under attend-left and attend-right conditions, and shaded backgrounds show the associated standard errors. Consistent across both types of chirp stimuli, tonic pre-stimulus amplitudes for both PAMs were larger when attention was directed toward as opposed to away from their side of the head. The noisier baselines in the waveforms elicited by the deviant chirps can be attributed to the much fewer single-trials included in the averages to these infrequent stimuli. Modulations of the PAM reflex were not analyzed in this study and will be presented elsewhere.

Figure 4 shows the averaged waveforms across participants for each combination of PAM location and attend-left/right conditions that resulted from the time-locked analysis (see Materials and Methods, section Analysis of tonic effects in time-locked PAM activity), separately for PAM responses time-locked to standard and deviant chirps. These waveforms indicated that pre-stimulus amplitude levels were virtually identical preceding responses that were time-locked to standard and deviant chirps. This finding strongly supports the hypothesis that the generated stimulation sequences were appropriately randomized, requiring participants to direct endogenous attention in a sustained, anticipatory manner. Most importantly, the time-locked waveforms for both types of chirps indicated that pre-stimulus amplitudes for both PAMs were larger when attention was directed toward as opposed to away from their side. To statistically analyze this effect of listening condition, planned contrasts (within-subjects *t*-tests) were performed by averaging the waveforms according to whether the PAM locations and attended locations were on the same or opposite sides and comparing the mean pre-stimulus amplitudes between −100 and 0 ms for these two conditions. Fully consistent with the tonic PAM amplitude analysis shown in Figures 2 and 3, these pre-stimulus differences were confirmed to be highly significant for standard [*t*(16) = 5.04, *p* = 1.21 · 10^−4^] as well as deviant stimuli [*t*(16) = 6.00, *p* = 1.88 · 10^−5^], thereby demonstrating that the observed attention effects resulted from continuously elevated muscle tone in the PAM ipsilateral to the attended side and were not dependent upon amplitude differences in time-locked post-stimulus responses. The PAM reflex waveforms are evident in the interval of 5–30 ms after the chirp offset. However, detailed analyses of attention effects on the PAM reflex are beyond the scope of the present paper and will be presented elsewhere.

## Discussion

The present study investigated the effects of endogenous attention on tonic activity of the posterior auricular muscle (PAM) during a dichotic selective listening task. By presenting randomized stimulation sequences with discrete, intermittent stimuli, this experiment was designed to analyze PAM modulations that could arise during a sustained, anticipatory mode of endogenous attention. The key finding was that ipsilateral PAM tone was elevated when sounds in one ear were selectively attended. This observation accords with the results of Strauss et al. (2020), who observed similar ipsilateral modulations during dichotic listening to continuous speech. Consequently, the present findings strongly suggest that this tonic effect of endogenous auditory attention on the vestigial auriculomotor system does not depend upon the inherent predictability of natural speech stimulation and is generalizable across a wide range of selective listening scenarios with lateralized stimuli.

The main analyses of the reported attention effects on tonic PAM activity were carried out on RMS envelopes of the ongoing EMG activity (Fig. 2) that were segmented without regard to stimulus events. Importantly, however, the same pattern of results was also observed for pre-stimulus amplitudes of rectified PAM responses that were time-locked to standard and deviant chirp stimuli (Fig. 4), thereby removing any influences from possible attention effects on PAM reflex amplitudes. This indicates that the increases in tonic PAM activity when same-side sounds were selectively attended were independent of the PAM reflex and consisted of purely sustained activity. It is important to note that this independence could not be established using continuous EMG recordings of rectified PAM activity, as was done in our primary analysis of tonic activity (Figs. 2 and 3) and by Strauss et al. (2020). Thus, the present results further reinforce the interpretation of Strauss et al. (2020) regarding their observations as modulations of sustained peri-auricular activity. As the pre-stimulus amplitudes in time-locked PAM responses to standard and deviant stimuli were virtually identical, the results additionally verify that the stimulation sequences were unpredictable, thereby confirming that the tonic modulations of PAM activity were a consequence of a sustained, anticipatory mode of endogenous attention.

In addition to the attention effects discussed above, tonic PAM activity declined monotonically over the course of the listening trials, for both PAM locations and listening conditions. This finding accorded with previous studies that revealed similar decrements over time during dichotic presentation of ongoing speech (Strauss et al., 2020). While the precise mechanism behind this time decay phenomenon remains elusive, it suggests that lateralized tonic PAM activity may be elevated during an initial adjustment period at trial onset and decreases as participants adopt a listening strategy that enables them to direct attention toward an auditory stream with less effort. Whereas this general decline of tonic PAM activity over time was previously found to be independent of whether ongoing speech stimuli were presented at the front or back of the listener, tonic activity of the AAM and SAM showed reversed patterns during stimulus presentation from the back (Strauss et al., 2020). These observations indicate that the auriculomotor system, despite its diminished anatomical and functional expression (Hackley, 2015), operates in a complex and nuanced manner. Along these lines, previous studies have demonstrated modulations of peri-auricular EMG activity obtained in different experimental scenarios, including, e.g., non-selective attentional paradigms (Stekelenburg and van Boxtel, 2002; Strauss et al., 2020; Schroeer et al., 2023, 2024), situations of effortful listening (Schroeer et al., 2025), and emotionally arousing situations (Benning et al., 2004; Benning, 2011, 2016).

From a more general perspective, the present observations reinforce the hypothesis that a vestigial form of a pinna-orienting system is still persistent in humans, and that auriculomotor activity could represent an attempt to orient the pinna to optimize sensitivity toward behaviorally important stimuli (Hackley, 2015; Strauss et al., 2020). More specifically, substantial evidence has accumulated to indicate that EMG activity recorded from these peri-auricular muscles comprises several distinct components. For the PAM, these components can be clearly separated into: (1) the tonic activity that was of major interest in the present experiment and the selective listening to speech study of Strauss et al. (2020), (2) the event-related PAM reflex at short latencies (5–30 ms) that was analyzed in the selective attention study of Hackley et al. (1987) and observed (but not analyzed) in the present study, and (3) event-related transient PAM responses at longer latencies of approximately 70 ms or later after stimulus onset that have been recorded in response to naturalistic auditory stimuli presented at unpredictable locations (Strauss et al., 2020; Schroeer et al., 2023, 2024). Notably, PAM response illustrations in previous studies that have reported these long-latency response components seem to be missing the earlier PAM reflexes (Strauss et al., 2020; Schroeer et al., 2023, 2024). This indicates that event-related auriculomotor responses could be dependent on the type of auditory stimuli as well as the (un)certainty of stimulus locations in space. Investigating these influences on PAM responses as well as possible interactions between the above-mentioned response components could provide a fruitful area for future research.

Apart from contributing to a deeper understanding of the different mechanisms that modulate auriculomotor activity, the present study also reinforces recent proposals that have advocated the integration of auriculomotor activity in the design of smart hearing aids (Liugan et al., 2018; Strauss et al., 2020; Schroeer et al., 2023). Specifically, the tonic PAM amplitude could provide a straightforward means for indicating a person’s lateralized focus of auditory attention in dual-speaker scenarios with spatially separated speakers. This information could then be leveraged to enhance the SNR for sound sources emanating from this position via intelligent noise reduction algorithms. Similar benefits may also be expected in scenarios of three or more competing speakers, in which the joint analysis of tonic PAM activity and appropriate EEG features, such as the degree of synchronization between cortical activity and the individual speech envelopes (Schäfer et al., 2018; Mai et al., 2022, 2025), could stabilize the identification of the attended stream. As auriculomotor activity can be reliably detected inside the ears (Schroeer et al., 2023), the tonic PAM amplitude may also be recorded via appropriate wireless in-ear systems (Kaveh et al., 2024), which would make it a promising candidate for use in situations that require robust decoding of the lateralized focus of auditory attention with a maximum degree of mobility.

## Synthesis

Reviewing Editor: David Schoppik, New York University Grossman School of Medicine Decisions are customarily a result of the Reviewing Editor and the peer reviewers coming together and discussing their recommendations until a consensus is reached. When revisions are invited, a fact-based synthesis statement explaining their decision and outlining what is needed to prepare a revision will be listed below. The following reviewer(s) agreed to reveal their identity: NONE. Note: If this manuscript was transferred from JNeurosci and a decision was made to accept the manuscript without peer review, a brief statement to this effect will instead be what is listed below.

The revised manuscript addresses the Reviewers' concerns and is stronger for the effort.
